# An effective fusion model for seizure prediction: GAMRNN

**DOI:** 10.3389/fnins.2023.1246995

**Published:** 2023-08-22

**Authors:** Hong Ji, Ting Xu, Tao Xue, Tao Xu, Zhiqiang Yan, Yonghong Liu, Badong Chen, Wen Jiang

**Affiliations:** ^1^Shaanxi Provincial Key Laboratory of Fashion Design Intelligence, Xi'an Polytechnic University, Xi'an, China; ^2^School of Software, Northwestern Polytechnical University, Xi'an, China; ^3^Xijing Hospital, Fourth Military Medical University, Xi'an, China; ^4^Institute of Artistic Intelligence and Robotics, Xi'an Jiaotong University, Xi'an, China

**Keywords:** EEG, spatial temporal feature, seizure prediction, attention module, GAMRNN

## Abstract

The early prediction of epileptic seizures holds paramount significance in patient care and medical research. Extracting useful spatial-temporal features to facilitate seizure prediction represents a primary challenge in this field. This study proposes GAMRNN, a novel methodology integrating a dual-layer gated recurrent unit (GRU) model with a convolutional attention module. GAMRNN aims to capture intricate spatial-temporal characteristics by highlighting informative feature channels and spatial pattern dynamics. We employ the Lion optimization algorithm to enhance the model's generalization capability and predictive accuracy. Our evaluation of GAMRNN on the widely utilized CHB-MIT EEG dataset demonstrates its effectiveness in seizure prediction. The results include an impressive average classification accuracy of 91.73%, sensitivity of 88.09%, specificity of 92.09%, and a low false positive rate of 0.053/h. Notably, GAMRNN enables early seizure prediction with a lead time ranging from 5 to 35 min, exhibiting remarkable performance improvements compared to similar prediction models.

## 1. Introduction

Epilepsy, also known as “fits” or “the falling sickness,” is a chronic neurological disorder in which sudden, abnormal electrical activity in the brain causes disruptions in its normal functioning (Artameeyanant et al., 2017). It is estimated that almost 65 million people worldwide have epilepsy, which accounts for ~1% of the global population (Bou Assi et al., 2017). The clinical manifestations of epilepsy are complex and varied, with symptoms ranging from motor, sensory, autonomic, and cognitive disturbances. While certain medications can help reduce the frequency of epileptic seizures, they are not always effective and may lead to serious side effects, threatening to the patients' daily lives and overall safety. Therefore, developing a reliable algorithmic model for predicting epileptic seizures, which can provide early warning and preventive measures, is paramount for the patients' survival.

**Figure 1:**
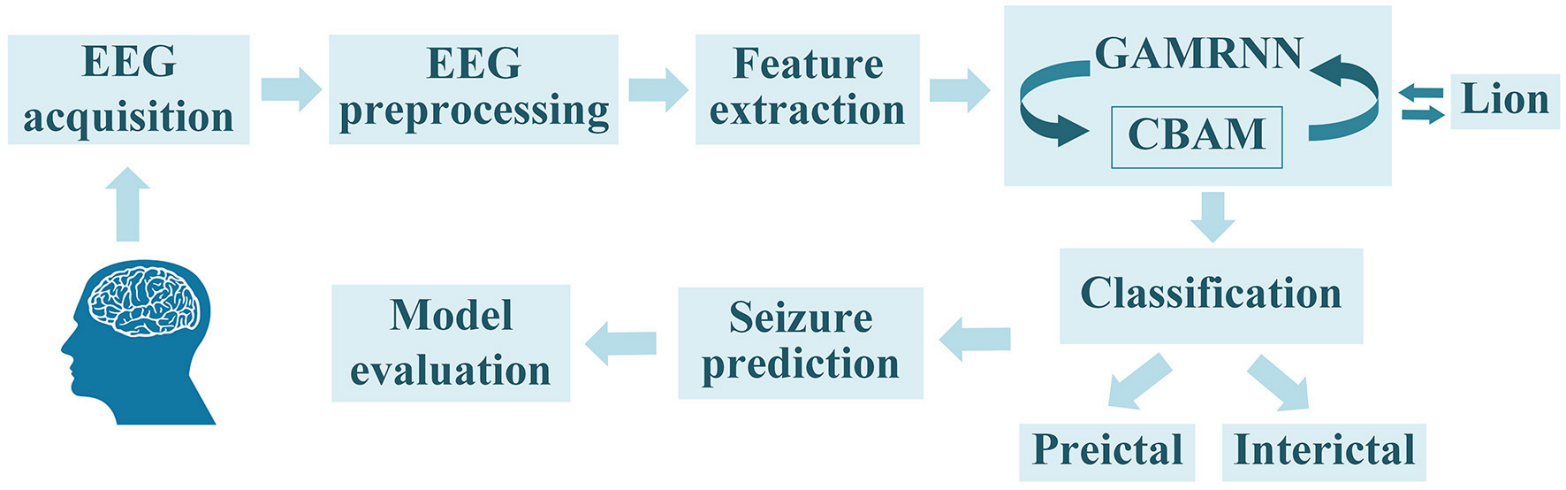
The overall seizure prediction method flow, as shown in the figure, includes EEG signal acquisition, EEG preprocessing, feature extraction, training the EEG graphs using the GAMRNN epileptic seizure prediction model augmented with the CBAM module, optimizing the trained model through the Lion optimization algorithm, partitioning the EEG signals into interictal signals and preictal signals, and ultimately predicting seizures and assessing the model's performance.

As an epileptic seizure begins, brain activity transitions from one state to another, accompanied by significant changes in the brain's electrical signals. Electroencephalography (EEG) is an effective method for monitoring the waveform changes in brain electrical signals during epileptic seizures. The EEG during a seizure can be categorized into four main states: preictal (a period before the onset of a seizure), ictal (a period during the seizure), postictal (a period following the seizure), and interictal (a period when the brain is not experiencing a seizure; Natu et al., 2022). Experienced experts can discern distinct states of epileptic seizure electroencephalogram (EEG) signals through observation. Nonetheless, the manual segmentation process of epileptic seizure signals is often laborious and time-consuming, necessitating graphologists with a high level of technical proficiency. Hence, its practical applicability is inherently challenging. The primary task in epileptic seizure prediction is to accurately extract features from EEG signals during the seizure period and differentiate them based on distinctive characteristics to separate preictal and interictal signals. This enables the prediction of the potential timing of a seizure, providing early warnings to patients and facilitating the implementation of intervention and remedial measures to minimize the impact of seizure episodes on patients. Throughout the course of an epileptic seizure, the importance of different EEG channels in seizure prediction research varies. Thus, there are challenges in selecting informative channels to extract more valuable feature information that ultimately helps improve the performance of seizure prediction models. Based on prior work, our proposed method for epileptic seizure prediction primarily encompasses the following steps: EEG signal acquisition, EEG preprocessing, feature extraction, model learning and training, classification of interictal and preictal data segments, seizure prediction, and model evaluation. During the model training, we incorporated the Convolutional Block Attention Module to enhance the model's attention to important channels and valuable feature information. Additionally, we utilized the Lion optimization algorithm for further optimization of the model training, ultimately improving seizure prediction performance, as illustrated in Figure 1.

In order to extract features that can effectively differentiate between pre-ictal and interictal EEG, prior researchers have attempted various methods. The most commonly used features include wavelet energy, power spectral density, phase locking value, permutation entropy, and fractal dimension value (Li et al., 2013; Joshi et al., 2014; Khalid et al., 2015; Zhang et al., 2020). Fei et al. (2017) used an improved largest Lyapunov exponent algorithm to better characterize the chaotic dynamical characteristics of EEG signals during epilepsy seizures, and the results showed that the improved algorithm had higher accuracy in identifying pre-seizure signals. Raghu et al. (2019) proposed a continuous decomposition index feature, which was proven to have a significant enhancement trend during epilepsy seizures so that the epilepsy seizure could be predicted in the pre-seizure period based on its changes. In addition, some studies use methods such as CSP transformation, principal component analysis, and autoregressive models to extract frequency or spatial domain features during epilepsy seizures (Büyükçakır et al., 2020). Due to the subjective selection of feature information, which may result in feature redundancy or the absence of crucial features, some researchers have proposed feature selection algorithms to select the optimal feature information (Karthick et al., 2018). Varatharajah et al. (2017) developed a scalp electroencephalogram (EEG) processing pipeline and introduced a seizure prediction method. The research findings indicate that the performance of the proposed prediction algorithm surpasses that of the baseline algorithm on the tested feature set. Bandarabadi et al. (2015) used an amplitude distribution-based feature selection algorithm; the study showed that this algorithm could also improve the accuracy of epilepsy prediction. After feature information extraction, the next step is to perform binary classification on the EEG signals. Yang et al. (2018) proposed a data analysis modeling method, and research showed that a seizure prediction system based on support vector machines could achieve robust preictal and interictal signals prediction. Yuan et al. (2017) utilized the diffusion distance measure and employed the Bayesian linear discriminant analysis to identify the periodicity of pre-seizure EEG signals, achieving high sensitivity and low false alarm rate. In addition, various methods have been used in seizure detection tasks, such as extreme learning machines, linear discriminant analysis, decision trees, random forest, etc. (Song et al., 2012; Rasekhi et al., 2013; Hussain, 2018; Mohan et al., 2018).

With the significant advancements of deep learning techniques in fields such as computer vision, it has also started to be gradually employed in the research of epileptic seizure prediction (Yıldırım et al., 2018; Liu et al., 2019; Yu et al., 2020). Firstly, the prediction model based on Convolutional Neural Networks (CNN) can well capture the feature information of EEG data due to its characteristics of local connectivity, weight sharing, and downsampling in time and space. Shasha et al. (2021) partitioned the experiment into two phases. They computed the Pearson correlation coefficient of the EEG signals. Subsequently, they fed the resulting correlation matrix into a simplistic CNN model to perform binary classification between interictal and preictal states. This approach effectively minimized computational overhead and yielded an accuracy rate of 89.98% when evaluated on the CHB-MIT dataset. Hu et al. (2019) employed CNN as a feature extraction model and used support vector machines (SVM) as classifiers for analyzing electroencephalograms (EEG). Truong et al. (2018) used STFT to extract frequency-domain and time-domain information from EEG signals on a 30 s window and input the transformed spectrogram into the neural network for model training. The model was evaluated on the Freiburg, CHB-MIT, and American Epilepsy Society seizure prediction challenge datasets and could predict seizures from 30 to 5 min before the onset of seizures, substantiating the advantages and generalization abilities of CNN in the field of epileptic seizure prediction research for capturing EEG signal features.

Nevertheless, despite the impressive capability of CNNs in extracting spatial features from signals, they encounter significant limitations when it comes to capturing the temporal dynamics of the signals, which is crucial for identifying and predicting epileptic seizures. Recurrent neural networks (RNNs) can handle sequential data and are suitable for non-stationary time series signals such as EEG data, as they can directly learn from raw EEG data to preserve the maximum temporal feature information of the signal (Ghosh et al., 2017). However, as the depth of RNNs increases, problems such as gradient explosion or vanishing may occur, so researchers have proposed methods using improved RNNs such as Long Short-Term Memory (LSTM) and Gated Recurrent Unit (GRU). Tsiouris et al. (2018) employed a feature extraction methodology to extract raw EEG information and employed Long Short-Term Memory (LSTM) networks to generate prediction outcomes. Furthermore, the study evaluated the influence of different preictal windows on the assessment results. Impressively high sensitivity and specificity rates of 99.28% were achieved, along with a false alarm rate of 0.107/h. This experiment also confirmed the outstanding performance of LSTM in analyzing preictal EEG signals. Varnosfaderani et al. (2021) proposed an epileptic seizure prediction model based on a two-layer LSTM and Swish activation function. This structure performs feature extraction based on both time and frequency domains and uses the minimum distance algorithm as a post-processing step. The model achieved a sensitivity of 86.8%, prediction accuracy of 85.1%, and a low false positive rate of 0.147/h when evaluated on the Melbourne dataset, which indicates that LSTM performs at a comparable level to CNN in the research of epileptic seizure prediction and may even have a more significant advantage in capturing the temporal features of EEG signals.

Continuous efforts of previous studies have demonstrated that integrating temporal and spatial characteristics of EEG signals is essential for enhancing the efficiency of epileptic seizure prediction. Consequently, algorithms combining Convolutional Neural Networks (CNNs) and Recurrent Neural Networks (RNNs) have emerged to capture the crucial temporal and spatial feature information of EEG signals. Affes et al. (2019) proposed a Convolutional Gated Recurrent Neural Network (CGRNN) for seizure prediction and demonstrated that this model outperformed a CNN-only model in predicting seizures, achieving an average sensitivity of 89% and an average accuracy of 75.6% using a dataset from Boston Children's Hospital. Hu et al. (2020) developed a deep bidirectional long short-term memory (Bi-LSTM) network as a predictive model for epileptic seizure prediction. The experiments employed local mean decomposition (LMD) and statistical feature extraction techniques to capture essential features. The achieved sensitivity of the model was 93.61%, with a specificity of 91.85%. However, these models still face challenges in distinguishing useful signals from noise and irrelevant information, which may lead to reduced the performance of seizure prediction.

Since its introduction, the attention mechanism has been widely applied in various fields such as computer vision (Zhu et al., 2018) and natural language processing (Wu et al., 2019). This is due to its ability to allow neural network models to focus more on relevant information in the input while reducing attention to irrelevant information. Consequently, it has been applied in epileptic seizure prediction research to help models accurately capture useful temporal and spatial features in EEG signals. Concentrating on the most relevant EEG signals and disregarding noise and irrelevant information can improve the classification, and prediction performance of the models. Choi et al. (2022) proposed an ACGRU generalized prediction model that combines one-dimensional convolutional layers, gated recurrent unit layers, and attention mechanisms across patient paradigms to classify preictal and interictal data. Improved classification accuracy and predictive performance were achieved on the EEG dataset of epileptic patients from Eshan Medical Center Children's Hospital, surpassing the performance of the original model. Wang et al. (2022) proposed adding a channel attention module to their CNN-LSTM-based seizure prediction model to address the issue of equal weighting for each channel's feature map in traditional models, achieving an accuracy of 83.04% after training and improving the recognition rate during the correct seizure period. These experiments have consistently demonstrated that neural network models with incorporated attention modules exhibit superior performance in seizure prediction algorithms.

**Figure 2:**
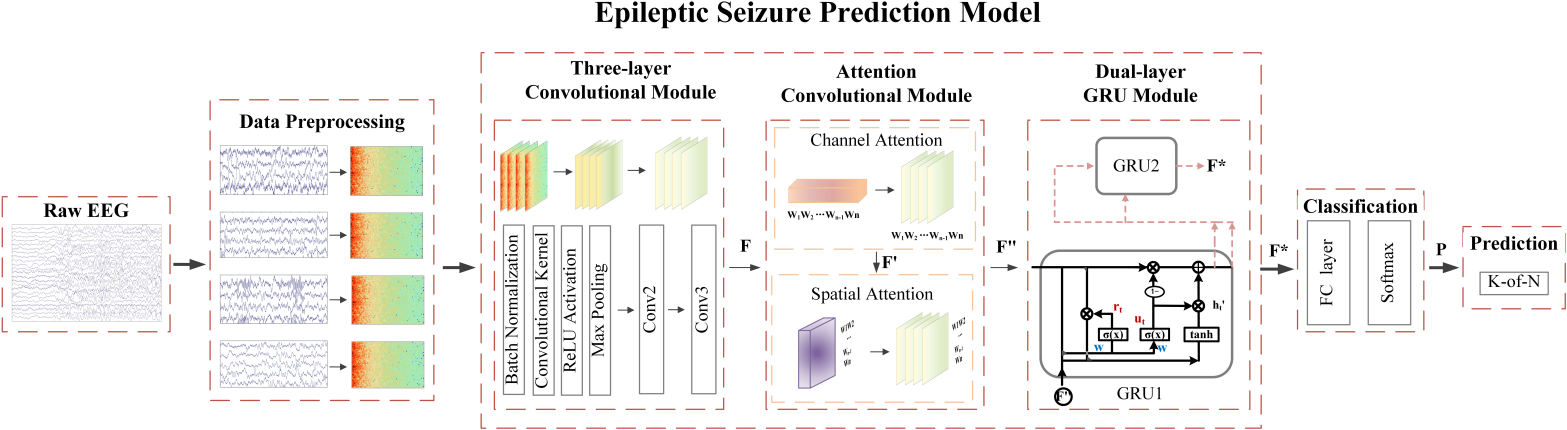
The overall workflow of the seizure prediction algorithm involves the following steps: During the data preprocessing stage, the raw EEG signals are transformed into two-dimensional time-frequency representations. These spectrograms are then fed into our proposed seizure prediction model. The initial feature extraction is performed by applying three layers of convolutional operations, resulting in the generation of a feature matrix denoted as *F*. Subsequently, the convolution attention module is applied to the obtained feature map, applying channel-wise and spatial weighting to produce *F*″. Next, the deep temporal feature extraction and modeling of the feature map are conducted using bidirectional gated recurrent units, resulting in F^*^. Following this, the data is passed through fully connected layers and subjected to a softmax function to perform binary classification, predicting the probability *P* of the model classifying the data into interictal and preictal states. Finally, post-processing is employed to make seizure predictions.

Attention modules contribute to the enhancement of predictive performance. They operate independently on either channel-specific or spatial-specific features of EEG signals. In addition, the convolutional attention module combines channel attention and spatial attention, facilitating concurrent processing of both channel and spatial information. This incorporation enables the model to comprehensively capture critical features across diverse channels and spatial dimensions, thereby elevating the accuracy and robustness of epileptic seizure prediction. The convolutional attention module (Woo et al., 2018) achieves the weighting operation on the channel and spatial information of the feature matrix through the stacking of blocks and attention modules. This process optimizes the relationship between different EEG channels and different spatial features automatically, enabling the model to focus more deeply on the essential signal features of the spatial structure of the EEG. Ultimately, it aims to optimize the performance of the model. On this basis, we propose an epileptic seizure prediction model with a graph attention module incorporating recurrent neural networks (GAMRNN) and use a novel optimization algorithm to train the model, combining multiple layers of convolution and double layers of GRU units to jointly extract the spatiotemporal features of the EEG, as shown in Figure 2. The main contributions of this research are as follows:

1. We propose a novel epileptic seizure prediction model, GAMRNN, which incorporates a convolutional attention module to focus on important channel and spatial information in EEG signals, enabling more effective capturing of spatio-temporal features.
2. We utilized the recently introduced Lion optimizer to optimize the model, thereby expediting the convergence rate of the network model training and facilitating the performance of the proposed model in epileptic seizure prediction.
3. Through ablation experiments on various combined models, we further validated the crucial roles of the Convolutional Block Attention Module and the Lion optimizer in epileptic seizure prediction tasks.

## 2. Materials and methods

### 2.1. Epileptic seizure prediction model

**Figure 3:**
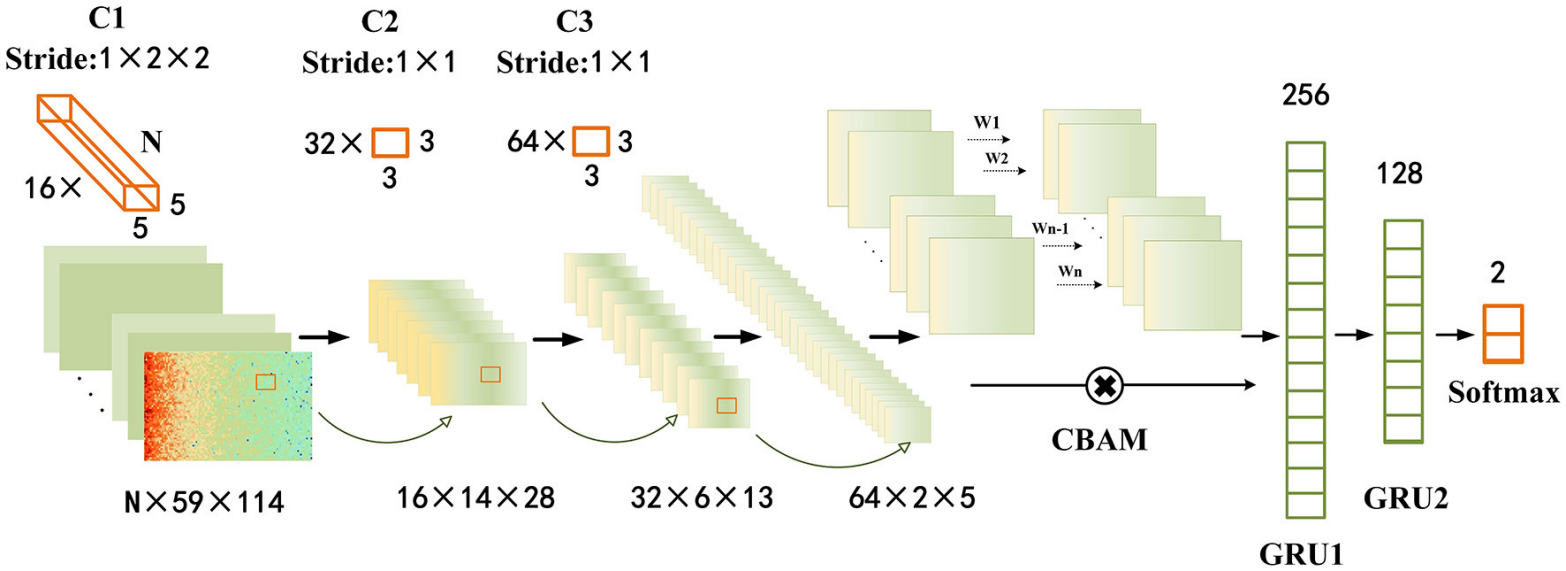
Epileptic seizure prediction model: the figure depicts the comprehensive framework of our proposed model for seizure prediction (GAMRNN). The input consists of 30-s windows of preprocessed EEG signals obtained through STFT transformation. The model begins with three convolutional blocks named C1, C2, and C3, serving as the initial feature extraction modules. Each block consists of a batch normalization layer, a convolutional layer with ReLU activation function, and a max pooling layer. C1 has 16 three-dimensional kernels of size *n* × 5 × 5, where *n* represents the number of channels in the original EEG signal, and the stride is 1 × 2 × 2. After the convolution operation, the results are passed through a ReLU activation function, followed by max pooling with a shape of 1 × 2 × 2 to perform downsampling. The operations in C2 and C3 are the same, with 32 and 64 convolutional kernels, respectively. The kernel size is 3 × 3, and the stride is 1 × 1. Both C2 and C3 also employ max pooling with a shape of 2 × 2 for downsampling. Next, the extracted feature maps are subjected to channel and spatial attention-weighted operations using the CBAM module. The input and output feature maps have the same shape of 64 × 2 × 5. Subsequently, the feature maps are flattened and reshaped, and inputted into the first gated recurrent unit (GRU) layer with 256 units, followed by a fully connected layer with sigmoid activation function. The output is then fed into the second GRU layer with 128 units, and finally through two fully connected layers with 2 units and softmax activation function for classification. Two dropout layers with a dropout rate of 0.5 are placed before the two fully connected layers.

Convolutional neural networks (CNNs) have been proven to possess certain advantages in capturing spatial features in data. In contrast, recurrent neural networks (RNNs) have been demonstrated to excel in capturing temporal features of data. Previous studies have also confirmed that combining both CNNs and RNNs is conducive to identifying the temporal and spatial dependencies of epileptic seizure EEG signals. This work employed a multi-layer convolutional neural network (CNN) combined with a two-layer gated recurrent unit (GRU) as the fundamental model for epileptic seizure prediction. To extract more critical temporal and spatial feature information from important channels and spatial regions, we propose to incorporate the CBAM scheme into the base model and name it Graph Attention Module with Recurrent Neural Networks (GAMRNN) for the overall architecture of the seizure prediction model, as illustrated in Figure 3. The CNN is responsible for extracting spatial features from EEG signals, the CBAM module selectively attends to relevant information from input feature maps with larger weights in channels and spatial feature points, and the GRU layer is used to capture the temporal dynamics in the EEG feature map.

#### 2.1.1. Convolutional feature extraction module

Given the limited size of the training dataset and for the sake of model simplicity, we employed a straightforward and shallow three-layer CNN architecture. The model consists of three-layer convolutional blocks for feature extraction. Each block comprises a batch normalization with a RELU activation function, followed by a max pooling layer. To ensure uniform input distribution across layers, batch normalization is applied between each layer, irrespective of the preceding layer's operations. The convolutional layer employs 16 kernels of size *n*×5 × 5, 32 kernels of size 3 × 3, and 64 kernels of size 3 × 3, where *n* represents the number of channels in the EEG signal. The stride for each kernel is 1 × 2 × 2, 1 × 1, and 1 × 1, respectively. In order to enhance the performance of the epilepsy seizure prediction task and mitigate the risk of overfitting, L2 regularization terms were incorporated into each convolutional layer. This regularization technique promotes weight values to be smaller and encourages a balanced distribution of weights. Consequently, it improves the convergence speed and stability of the model, thus aiding in accurate epilepsy seizure prediction. The max pooling layer has a size of 2 × 2, which is used to reduce the number of computations and prevent overfitting during model training. After the initial feature extraction, a feature map of size 64 × 2 × 5 is obtained.

#### 2.1.2. Attention enhancement module

In a seizure prediction system, focal epileptic EEG signals originate from one or multiple scalp electrodes, propagate and gradually spread to multiple electrodes and brain regions. They are characterized by overlapping and interfering waveforms. Some electrodes may be located in more relevant or active pathological areas, while others may be in less related or less active brain regions. Some electrodes may be located in more relevant or active pathological areas, while others may be in less relevant or less active brain regions. Therefore, the importance of signals varies among electrodes. In this case, the attention module can assign different weights to different electrodes and features, allowing the system to focus on essential electrodes or features.

A previous study has investigated using the CBAM module, integrated after batch-normalized long short-term memory (Ma et al., 2021) (BNLSTM) networks, for end-to-end seizure prediction based on raw EEG data. By introducing the attention mechanism, the system may capture the key channels and features more related to seizure events, thereby improving prediction performance. In this experiment, we placed the CBAM module after the three convolutional layers, allowing feature selection to be performed on the already processed feature maps. This approach ensures that the selected features are more accurate and representative, thereby enhancing the performance and effectiveness of the seizure prediction system.

**Figure 4:**
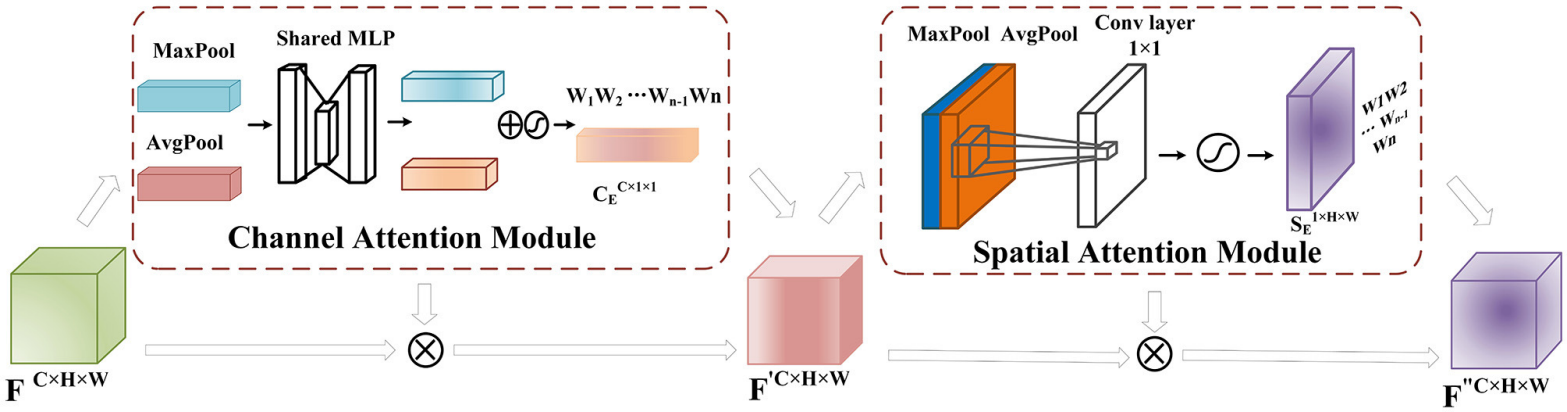
CBAM Module: After the feature extraction, the feature matrix *F*∈*R*^*C*×*H*×*W*^ is obtained. In the channel attention module, global and max pooling operations are applied to *F* to obtain a *C*×1 × 1 tensor. This tensor is then passed through two fully connected layers with a ReLU activation function, reducing the channel dimension to one-fourth of the original number and then restoring it to the original dimension to obtain the feature matrix mapping. The mapping is combined with *F* using a sigmoid activation function to assign weights $C_{E}\left(F\right)\in R^{C\times 1\times 1}$ to each input channel, representing channel importance levels. The obtained weight matrix is multiplied by the original input feature matrix to obtain *F*′. In the spatial attention module, *F*′ undergoes global average and max pooling operations, and the resulting tensors are stacked and processed with a 1 × 1 convolution. The sigmoid activation function maps the spatial feature weights to the range [0,1], obtaining the weights $S_{E}\left(F\right)\in R^{1\times H\times W}$ that represent the importance of each feature point. These weights are multiplied by *F*′ to obtain the weighted feature map *F*″.

CBAM consists of two modules, namely Channel Attention (Sun et al., 2019) and Spatial Attention (Chen et al., 2017), as shown in Figure 4. The feature map obtained after the convolution layer has the shape *F*∈*R*^*C*×*H*×*W*^ (where *C* is the number of channels and *H* and *W* are the height and width of the feature map obtained after convolution). For each channel, we set the convolution module as a 2D convolution kernel, and the feature map obtained through channel attention is *C*_*E*_, while that obtained through spatial attention is *S*_*E*_.

(a) Channel attention weighting mechanism

The convolutional operation produces a feature map *F*∈*R*^*C*×*H*×*W*^, where *C* denotes the number of channels, and *H* and *W* refer to the height and width of the feature map. This feature map is initially fed into the Channel Attention module as part of the CBAM module. Channel Attention compresses the feature map along the channel dimension and calculates weight coefficients for each channel. The output is a feature map with weight coefficients, where the dimension of the feature map remains the same as that of the input feature map. In order to improve computational efficiency, the input feature map is globally max-pooled and averaged-pooled to compress the feature map. The pooling resulted in obtaining two different feature descriptions that represent the spatial background features of the data. A channel-wise feature map of size $C_{E}\left(F\right)\in R^{C\times 1\times 1}$ is obtained through a shared fully connected layer. The two obtained feature matrix mappings are added and passed through a sigmoid activation function to assign proper weights (between 0 and 1) to each input channel C. Finally, the weight matrix is multiplied by the input feature layer. Although the channel attention module assigns weights to the channels of the feature matrix obtained through convolutional operations, it represents the reorganization and integration of the original EEG electrode channels, which implies that the channel attention module assists the model in extracting more important channel feature information from the EEG signals.

In this study, the channel attention module reallocates the importance and correlation of each channel in the EEG signal by generating weight coefficients for each channel based on the convolutional operations of the EEG electrode leads. The specific operation is shown in the formula below:


(1) $$\begin{array}{l} C_{E}\left(F\right)=\text{sigmoid}\left(\text{Conv}2\text{D}\left(\text{MaxPool}\left(F\right)\right)+\text{Conv}2\text{D}\left(\text{AvgPool}\left(F\right)\right)\right) \end{array}$$

(2) $$\begin{array}{l} F^{{\;}^{\prime }}=C_{E}\left(F\right)\times F \end{array}$$

(b) Spatial attention weighting mechanism

The spatial attention further extracts features from EEG data at the convolutional level, aiming to preserve the spatiotemporal information of EEG signals as much as possible. The input feature map *F*′∈*R*^*C*×*H*×*W*^ undergoes max pooling and average pooling operations at each feature point along the spatial dimensions. Then, the two results are stacked along the channel dimension. A 1 × 1 convolutional layer is applied to adjust the channel dimension to 1, and a sigmoid activation function is used to obtain weight values (between 0 and 1) for each feature point on the feature map. Finally, the weight matrix $S_{E}\left(F^{{\;}^{\prime }}\right)\in R^{1\times H\times W}$is multiplied by the original feature map to obtain the feature map *F*″. The convolutional layer adaptively learns features for each channel input, enabling the network to focus more on meaningful features in the signal and improve the accuracy of seizure prediction. The specific process is shown in the following formula:


(3) $$\begin{array}{l} S_{E}\left(F^{{\;}^{\prime }}\right)=\text{sigmoid}\left(\text{Conv}\left(\text{concat}\left(\left[\text{MaxPool}\left(F^{{\;}^{\prime }}\right),\text{AvgPool}\left(F^{{\;}^{\prime }}\right)\right]\right)\right)\right) \end{array}$$

(4) $$\begin{array}{l} F^{{\;}^{\prime \prime }}=S_{E}\left(F^{{\;}^{\prime }}\right)\times F^{{\;}^{\prime }} \end{array}$$

#### 2.1.3. Temporal modeling and classification module

The Gated Recurrent Unit (GRU; Chung et al., 2014) is an advancement over the Long Short-Term Memory (LSTM) model, offering a more streamlined architecture. It incorporates two gate mechanisms to regulate the flow and forgetting of preceding temporal information, effectively addressing the issue of vanishing gradients encountered in recurrent neural networks. Moreover, GRU exhibits enhanced capability in capturing long-term dependencies inherent in sequential data, making it well-suited for analyzing time-series signals. In our study, we employ a dual-layer GRU network to comprehensively analyze the extracted feature matrix *F*″, which allowed us to delve deeper into the temporal features of the electroencephalography (EEG) signals associated with seizure activity F^*^, thereby facilitating a more precise and accurate classification.

Specifically, in the GRU module, the hidden state *h*_*t*−1_ represents the temporal information from the previous time step, while *x*_*t*_ represents the current time step's input feature matrix. This study defines the time steps based on the sequential order of the input feature matrix *F*″. The influence of the previous hidden state *h*_*t*−1_ on the current time step is controlled by the reset gate *r*_*t*_, as shown in the following formula:


(5) $$\begin{array}{l} r_{t}=\text{sigmoid}\left(h_{t-1}W_{rh}+x_{t}W_{rx}+b_{r}\right) \end{array}$$

where *W*_*rh*_ and *W*_*ry*_ represent the weight matrices of the reset gate, and *b*_*r*_ is the bias matrix with a size equal to the number of hidden units *nh*. Moreover, the update gate $u_{t}\in R^{1\times nh}$ is responsible for controlling the balance between the previous hidden state and the current input at each time step, determining the extent to which the previous hidden state is retained and fused with the current input feature.


(6) $$\begin{array}{l} u_{t}=\text{sigmoid}\left(h_{t-1}W_{uh}+x_{t}W_{ux}+b_{u}\right) \end{array}$$

where weight matrices *W*_*uh*_ and *W*_*ux*_ represent the weights of the update gate, and *b*_*u*_ is equal to the number of hidden units *nh*. The temporary hidden state $h_{t}^{{\;}^{\prime }}$ at time step *t* is obtained by element-wise multiplication.


(7) $$\begin{array}{l} h_{t}^{{\;}^{\prime }}=\text{tanh}\left(h_{t-1}W_{hh}\times r_{t}+x_{t}W_{xh}\right) \end{array}$$

where weight matrices *W*_*hh*_ and *W*_*xh*_ are used, along with the hyperbolic tangent activation function tanh, to control the flow of information through the reset gate *r*_*t*_, which determines the degree to which the previous hidden state is retained. Finally, by utilizing the update gate *u*_*t*_, the new hidden state *h*_*t*_ is computed through a linear combination of the previous hidden state *h*_*t*−1_ and the current state $h_{t}^{{\;}^{\prime }}$, as shown in the following equation:


(8) $$\begin{array}{l} h_{t}=\left(1-u_{t}\right)\times h_{t-1}+u_{t}\times h_{t}^{{\;}^{\prime }} \end{array}$$

In summary, the distinctive feature of the dual-layer GRU module in predicting epileptic seizures lies in its effective integration of temporal information and modulation of information flow through gating mechanisms. The dual-layer GRU structure in this study consists of 256 and 128 units, with a dropout rate of 0.5 to mitigate overfitting. The first GRU layer learns temporal dependencies and sequential relationships of neighboring feature maps from *F*″. The second GRU layer captures deeper long-term dependencies and contextual information using the hidden state from the first layer, resulting in the feature matrix F^*^. By modeling and synthesizing temporal features, the dual-layer GRU module effectively utilizes the features extracted by the convolutional layer and CBAM module, enhancing the classification accuracy of seizure onset and interictal data and improving the prediction model's performance.

Following the GRU layers are two fully connected layers and two Dropout layers. The first fully connected layer has 64 neurons and uses the sigmoid activation function, taking the output of the Dropout1 layer as input. The second fully connected layer consists of 2 neurons, taking the output of the Dropout2 layer as input. Finally, the Activation layer is used to pass the final softmax output to the output layer of the model, completing the classification task.

### 2.2. Lion optimizer

During the model training process, we employed a recently proposed optimization algorithm called the Lion optimizer (Chen et al., 2023), developed by researchers from Google and UCLA. Unlike adaptive optimizers like Adam and SGD, the Lion optimizer only requires momentum tracking and utilizes symbolic operations to compute updates, leading to fewer hyperparameters and simpler computations. It has shown superior performance to traditional optimization algorithms when applied to deep learning models in tasks like image classification while accelerating the model training process. Thus, in our research, we introduced the Lion optimizer in the context of seizure prediction models and conducted comparative experiments with the Adam optimizer to assess its impact on model performance.

## 3. Experiment and results

In the experimental section, we firstly performed preprocessing on the EEG dataset, including splitting the raw data into 30-second windows, removing noise and artifacts, and transforming the EEG data into time-frequency spectrograms. Secondly, the data was partitioned into training and testing sets and fed into the model for training. Meanwhile, we utilized the Lion optimization algorithm to further optimize the model's training process. Thirdly, post-processing operations were applied to the obtained classification results to predict seizure occurrences, and various metrics were used to evaluate the model's seizure prediction performance. Finally, several ablation experiments were conducted to individually assess the impact of each component on the overall model's predictive performance.

### 3.1. Dataset

**Figure 5:**
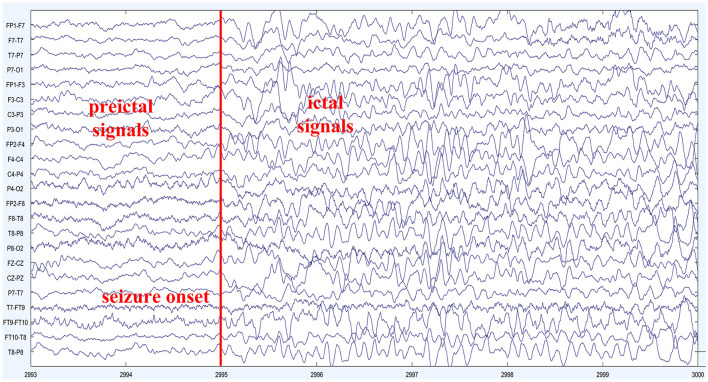
EEG signal of patient Chb01: the depicted figure showcases the scalp electroencephalographic (EEG) recordings obtained from subject Chb01 during a seizure episode. The onset of the seizure activity becomes evident at 2995 s, marked by a prominent escalation in EEG frequency and the emergence of complex waveforms characterized by irregularities and spikes. These distinct alterations in the EEG signal morphology signify the initiation of a seizure event, indicating a significant disruption in the underlying neural activity. The observed contrast in EEG patterns between the preictal and ictal periods highlights the pronounced impact of the seizure activity on the brain's electrical dynamics.

In this experiment, we utilized the CHB-MIT dataset to validate the seizure prediction performance of the proposed model (Goldberger et al., 2000). The dataset consists of scalp electroencephalogram (EEG) recordings from 23 pediatric epilepsy patients, collected through collaboration between the Massachusetts Institute of Technology (MIT) and Boston Children's Hospital. The EEG data were sampled at a rate of 256 Hz and acquired using 22 electrodes placed according to the international 10–20 system for EEG electrode placement. The dataset spans approximately 1136 hours of continuous EEG signal activity and includes 198 epileptic seizure events. The patients' ages range from 1.5 to 22 years. Detailed information about the dataset is provided in Table 1. The CHB-MIT public dataset provides expert annotations indicating the start and end times of seizure events. In this study, we define the interictal period as a time interval of at least 4 h before and after the seizure, following the standard proposed by Truong et al. (2018) for seizure prediction research, providing a reference for comparison with their method. Additionally, we excluded the cases with more than ten seizures in the dataset, as their seizure occurrences are too close in time and the prediction results are less meaningful for these patients. To facilitate comparison with related experiments, we evaluated the epilepsy seizure prediction model in detail using data from 13 patients. Figure 5 shows the EEG segments of a seizure event in patient chb01.

**Table 1:** Detailed information of seizure subjects in CHB-MIT dataset.

**Subject no**.	**Age**	**Gender**	**Records**	**Seizure onset**
Chb01	11	F	42	7
Chb02	11	M	36	3
Chb03	14	F	38	7
Chb04	22	M	42	4
Chb05	7	F	39	5
Chb06	1.5	F	18	9
Chb07	14.5	F	19	3
Chb08	3.5	M	20	5
Chb09	10	F	19	4
Chb10	3	M	25	7
Chb11	12	F	35	3
Chb12	2	F	24	21
Chb13	3	F	33	12
Chb14	9	F	26	8
Chb15	16	M	40	20
Chb16	7	F	19	10
Chb17	12	F	21	3
Chb18	18	F	36	6
Chb19	19	F	30	3
Chb20	6	F	29	8
Chb21	13	F	33	4
Chb22	9	F	31	3
Chb23	6	F	9	7

### 3.2. Data preprocessing

The raw EEG signals are characterized by a large quantity and continuous long duration, making them unsuitable for direct input into convolutional neural networks for feature extraction. Therefore, data preprocessing is required. Firstly, preictal and interictal data are extracted separately from the original EEG data. Subsequently, the data is splitted into 30-second windows, and the short-time Fourier transform (STFT) technique is employed to transform the raw EEG signals into two-dimensional spectrograms with frequency and time axes. The transformation helps retain crucial information from the original signals (Truong et al., 2019; Muhammad Usman et al., 2020). During data collection, the dataset is contaminated with 60 Hz power line noise. To address this issue, bandpass filtering is applied to remove frequency components between 57 Hz–63 Hz and 117 Hz–123 Hz, along with excluding the 0 Hz component.

Due to the uneven distribution of the two classes in the dataset, namely, the number of preictal data is significantly smaller than the number of interictal data in a single EEG recording of a seizure episode, it is likely that the model may not learn sufficient useful features due to the scarcity of one class during training, ultimately affecting the classification accuracy. To overcome this data imbalance issue, we employ the overlapping sampling technique along the temporal axis of the EEG signal, generating additional preictal samples using a sliding window of 30 s. After preprocessing, the spectrograms are fed into the GAMRNN model for feature extraction and classification. Through extensive training, the model learns the discriminative features of seizure EEG signals and performs sample classification into preictal and interictal states.

### 3.3. Experimental setting

In order to train the model and learn relevant features from the preprocessed dataset, it is necessary to partition the dataset into training and testing sets. Here, we employed the leave-one-out cross-validation method. For a subject with *N* occurrences of seizures in their data records, N-1 seizure interictal and preictal segments were concatenated as the training set, while the remaining occurrence of seizure interictal and preictal segments were used as the testing set. Furthermore, 75% of the training set data was utilized for training the model, while the remaining 25% was used as a validation set to assess the learning and training performance of the proposed model and prevent overfitting. We also incorporated an early stopping mechanism during the model training process. If the loss did not improve for ten consecutive epochs, the training was halted prematurely, and the model parameters with the best performance during training were saved. This approach aimed to minimize resource waste and training time.

The experiment was implemented on Python 3 using the Keras and TensorFlow frameworks. The training batch size was set to 64, and the number of epochs was set to 50. For Lion optimizer, the cross-entropy loss was used to compute the training loss. We set the hyperparameter β_1_ for exponential decay rate to 0.95, β_2_ to 0.98, learning rate η to 0.0001, and weight decay rate λ to 0.015 based on instructions of lion optimizer and our experiences.

### 3.4. Metrics for epileptic seizure prediction

**Figure 6:**
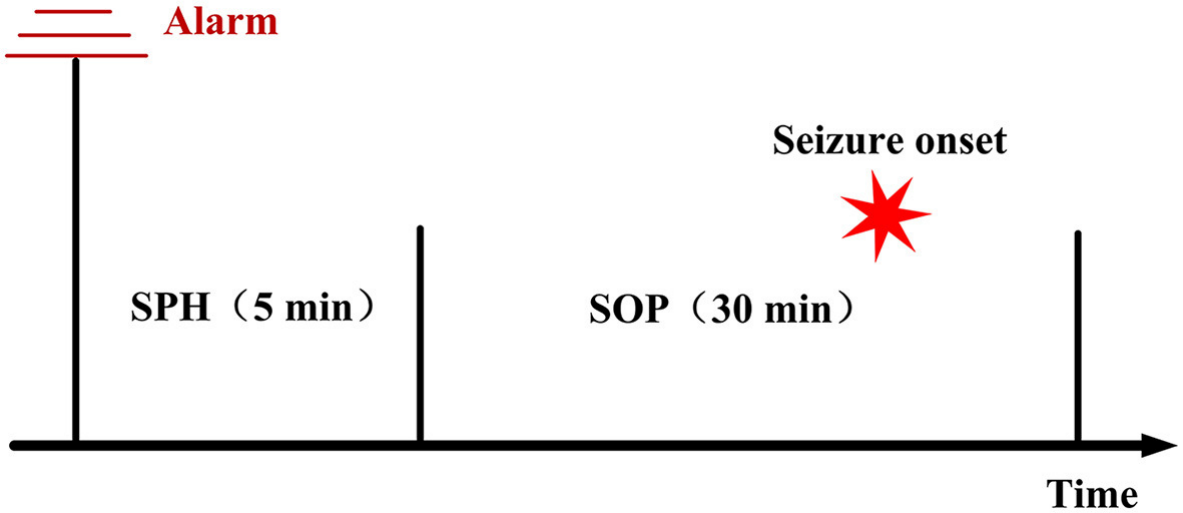
Diagram of the Seizure Prediction Period (SPH) and Seizure Onset Period (SOP): in the post-processing phase, a successful prediction of seizure onset by the seizure prediction model is defined as the absence of any seizure events during the SPH period following the onset alert, followed by the occurrence of one or more seizure events during the subsequent SOP period. When no seizure events occur during the SOP period following the onset alert, it is considered a false alarm.

Seizure prediction horizon (SPH) and seizure onset prediction (SOP) are two temporal periods used to evaluate the results of seizure prediction. SPH refers to the time interval from the onset of an alert to the expected seizure phase, while SOP represents the time span during which the seizure is anticipated to occur. A correct alert within the SPH serves to notify healthcare professionals and family members that a seizure is likely to happen within the subsequent SOP, enabling them to take timely measures. Consistent with Truong et al. (2018), this study sets the SPH to 5 min and the SOP to 30 min. The method for setting SPH and SOP is shown in Figure 6. The criterion for accurate prediction is the occurrence of at least one seizure event during the SOP period following the onset of the alert, while no seizures should occur within the SPH period. False alarms, on the other hand, refer to alerts issued in the absence of any seizures during the SOP period. To reduce false positives, a K-of-N post-processing method is employed (Truong et al., 2018), where an alert is triggered only when K seizure-like segments are identified within a continuous sequence of *N* segments. In this study, the parameters k = 8 and *n* = 10 are set, with predictions made every 30 s. Consequently, if more than 4 min of seizure-like segments are identified within a continuous 5 min data segment, an alert is issued.

The performance of the epilepsy seizure prediction model was evaluated using sensitivity (SEN), specificity (SPEC), accuracy (ACC), area under the curve (AUC), and false positive rate per hour (FPR/h) metrics. In typical binary classification tasks, sensitivity, specificity, and accuracy are calculated from the confusion matrix in statistics. Sample prediction can result in four possible scenarios: TP (True Positive), meaning the actual EEG signal data is preictal and the predicted result is also preictal; FP (False Positive), meaning actual interictal signal data is predicted as preictal signal data; TN (True Negative), meaning the predicted data is interictal signal data, and it is indeed interictal signal data; FN (False Negative), meaning actual preictal signal data is predicted as interictal signal data. Based on the confusion matrix, the following metrics can be calculated:


(9) $$\begin{array}{l} Sensitivity=TP/\left(TP+FN\right) \end{array}$$

(10) $$\begin{array}{l} Specificity=TN/\left(TN+FP\right) \end{array}$$

(11) $$\begin{array}{l} Accuracy=\left(TP+TN\right)/\left(TP+TN+FP+FN\right) \end{array}$$

The ROC (Receiver Operating Characteristic) curve is a graphical representation where the X-axis is the false positive rate (FPR), and the Y-axis is the true positive rate (TPR). Different TPR and FPR values can be obtained and plotted as an ROC curve by changing the classifier's threshold. The AUC (Area Under the Curve) is the area under the ROC curve, with a value between 0.5 and 1. A larger AUC indicates a better performance of the classifier.

## 4. Results

### 4.1. General results

Based on the same experimental settings, we conducted a performance evaluation of the GAMRNN seizure prediction model and compared it with the GCRNN prediction model. We selected the same 13 patient data from the CHB-MIT dataset for evaluation on both models. The experimental results are shown in Table 2. We observed and compared the classification and prediction performance of the two models from the aspects of accuracy, sensitivity, and false positive rate, taking the average values of all the subjects' experimental results. From the table, we can conclude that our proposed GAMRNN prediction model demonstrates better seizure prediction performance on most subjects' data, with an average accuracy of 91.73%, which is a 6.44% improvement over the CGRNN prediction model. The GAMRNN model achieved a sensitivity of 88.09% in correctly predicting seizures, showing an ~6% increase in sensitivity compared to the original model, which indicates that the model successfully captured 56 out of 64 seizures. After incorporating the attention convolution module and optimizing the model using the Lion optimizer in the CGRNN model, the false positive rate decreased from 0.2042 to 0.053/h. Except for patients Chb10 and Chb14, the false positive rate for seizures in other patients approached 0. The improvement in these evaluation metrics has significant practical implications for the daily life of epilepsy patients. Above results confirm that the proposed seizure prediction model can effectively distinguish between preictal and interictal EEG signal data, enabling accurate decisions on whether a seizure will occur in the later stage of the EEG signal, thereby greatly reducing the occurrence of false alarms for seizures.

**Table 2:** Seizure detection performance on the CHB-MIT dataset.

	**CGRNN**			**GAMRNN**		
**Patient**	**Accuracy**	**Sensitivity**	**FPR (/h)**	**Accuracy**	**Sensitivity**	**FPR (/h)**
Chb01	0.9337	0.8429	0.057	0.9455	0.9548	0
Chb02	0.9398	0.1611	0	0.9415	0.3652	0
Chb03	0.9313	0.6389	0	0.9417	0.8361	0
Chb05	0.6884	0.2867	0.3468	0.9088	0.88	0.0694
Chb09	0.9814	1	0	0.9945	0.9833	0
Chb10	0.6572	0.4056	0.566	0.7811	0.7389	0.2264
Chb13	0.8793	0.9967	0.2081	0.9141	0.9933	0.0694
Chb14	0.5178	0.8	0.7385	0.7711	0.8433	0.1846
Chb18	0.8525	0.6167	0.2041	0.904	0.9375	0.0408
Chb19	0.9827	0.7889	0	0.9893	0.9322	0
Chb20	0.8952	0.9733	0.1469	0.9544	0.99	0.098
Chb21	0.867	0.8125	0.3134	0.8903	1	0
Chb23	0.9615	0.9833	0.0752	0.9886	0.9967	0
Average	0.8529	0.7159	0.2043	0.9173	0.8809	0.053
Variance	0.0189	0.0723	0.0505	0.0047	0.0280	0.0054

However, due to various reasons, such as differences in the number of seizures, proximity to seizures, or patient-specific characteristics, the seizure prediction model may not achieve the same prediction performance for every patient. The variance calculated for various metrics of the two seizure prediction models indicates that our proposed model demonstrates greater stability in evaluating the 13 patient datasets compared to the baseline model. The comparative experiments also provide evidence that the attention modules indeed assist the seizure prediction model in focusing more on crucial regions within the feature maps. By incorporating channel and spatial dimensions, the attention modules enable the model to emphasize the essential spatiotemporal features in the EEG signal data while reducing attention to relatively less significant regions. As a result, the overall model performance for classifying two types of seizure EEG signals is enhanced, leading to significant improvements in accuracy, sensitivity, and false positive rate evaluations.

### 4.2. Results of ablation study

Our study conducted two sets of ablation experiments. The first set of experiments aimed to validate the performance enhancement of the GAMRNN model by adding the CBAM module and using the Lion optimizer. Specifically, the CBAM module and Lion optimizer were sequentially added to the model, and their performance on different datasets was compared and analyzed. The second set of experiments aimed to validate the individual effects of the Channel Attention Module (CAM) and Spatial Attention Module (SAM) when applied separately to the model. Additionally, we compared the combination module with the order of CAM and SAM switched to the CBAM module. The accuracy, sensitivity, and specificity results obtained from the two groups of ablation experiments are presented in Table 3.

**Table 3:** Ablation experimental results.

**Methods**	**Accuracy**	**Sensitivity**	**Specificity**
GAMRNN (CAM only)	85.71	76.32	86.27
GAMRNN (SAM only)	85.53	76.59	85.59
GAMRNN (Lion only)	87.47	76.17	88.75
GAMRNN (CBAM and Adam)	90.03	83.75	91.52
GAMRNN (CBAM and Lion)	91.73	88.09	92.09

GAMRNN (CAM only) and GAMRNN (SAM only):We incorporated Channel Attention Module (CAM) and Spatial Attention Module (SAM) separately into the model to assess the individual impacts of these attention mechanisms on model performance. Specifically, when CAM or SAM was added independently to the model, the accuracy remained similar. However, there was approximately a 6% decrease compared to the model using the combined attention mechanism CBAM. Moreover, sensitivity and specificity were lower than the Convolutional Attention Module. These results indicate that utilizing a single attention mechanism alone has a limited impact on the performance of the seizure prediction model. However, the predictive performance was significantly enhanced when employing the Convolutional Attention Module that integrates both CAM and SAM and applies them jointly to the model. Additionally, we conducted experiments by interchanging the order of the attention modules (first applying SAM and then CAM) and combining them in the model. While there was a slight improvement in accuracy and specificity, it was not as pronounced as the original CBAM combination, suggesting that the order of combining attention modules within the Convolutional Attention Module has the most significant impact on enhancing the model's performance. In conclusion, the Convolutional Attention Module plays a more prominent role in improving the seizure prediction model than individual CAM and SAM, and the specific order of combining CAM and SAM within the Convolutional Attention Module has the most significant influence on model performance enhancement.

GAMRNN (Lion only): This model is derived from the proposed model by removing the CBAM module, allowing for the evaluation of the epileptic seizure prediction performance without the attention convolutional module. The experimental results demonstrate that the model without CBAM exhibits a significant performance decrease in accuracy, sensitivity, and specificity compared to the proposed prediction model. Specifically, the classification accuracy of interictal and preictal data decreased from 91.73 to 87.47%. The sensitivity of correctly identifying preictal data decreased from 88.09 to 76.17%, and the specificity of correctly identifying interictal data also decreased by 3.34%. These changes in results indicate the crucial role of the CBAM module in the proposed epileptic seizure prediction model, as the model without CBAM shows a significant decrease in classification performance. Therefore, we hypothesize that the inclusion of CBAM in the model allows for further attention to be given to essential channels and spatial feature points within the feature maps after the initial three-layer convolutional feature extraction, thereby aiding the model in focusing on extracting more crucial feature information and enhancing the classification and prediction performance of the model.

GAMRNN (CBAM and Adam): This model is obtained by removing the Lion optimizer from the proposed model and using the Adam optimizer, which is the same as the baseline model, to observe its impact on model training. A comparison reveals that this model also experiences a corresponding decrease in performance in various aspects, although the decrease is not particularly significant. For instance, the average accuracy of the model without the Lion optimizer is only reduced by ~1.70%, the sensitivity is reduced by ~4.34%, and the specificity is reduced by 0.57%. During model training, a visual inspection indicates that each epoch takes ~1–2 s less than the Adam optimizer model. This suggests that the Lion optimizer accelerates the training process and effectively reduces the training loss of the model, thereby enhancing the stability of correct seizure prediction. In summary, the Lion optimizer plays a role in performance evaluation and training for epileptic seizure prediction research tasks. It also lays the foundation for utilizing the Lion optimization algorithm in more complex studies, offering more possibilities for training models in epileptic seizure prediction research.

**Figure 7:**
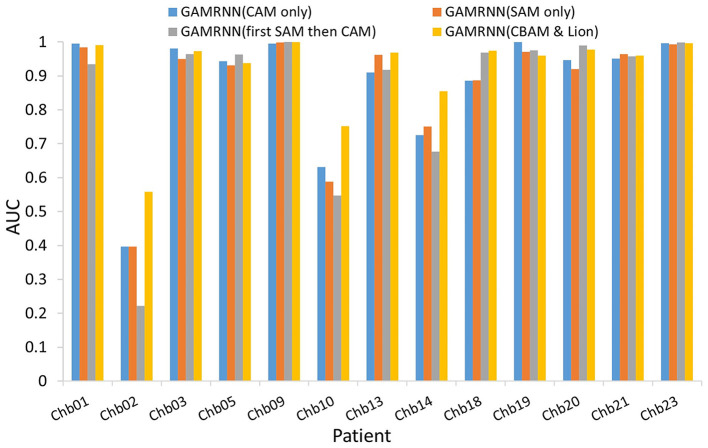
Comparison of AUCs with different attention mechanisms added to the models: the bar chart depicts the AUC evaluation results of four models, namely, GAMRNN (CAM only), GAMRNN (SAM only), GAMRNN (first SAM then CAM), and GAMRNN(CBAM and Lion), on 13 patient datasets. The comparison reveals that the proposed GAMRNN model with attention convolutional modules added in the normal sequence exhibits the most distinct and superior classification performance compared to the other three models.

**Figure 8:**
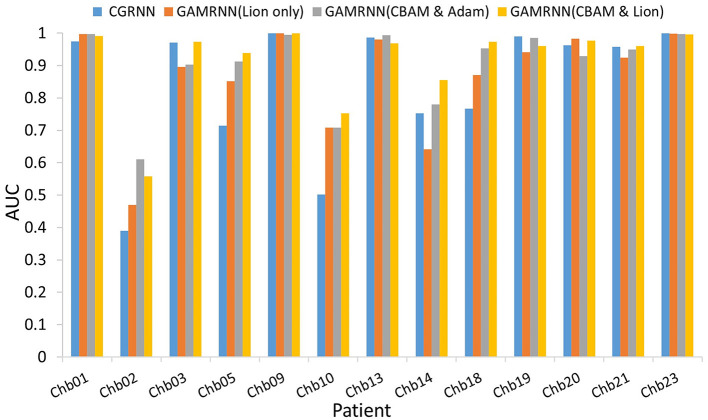
Comparison of AUC among different models: the figure depicts the AUC evaluation of four epilepsy seizure prediction models, namely CGRNN, GAMRNN (Lion only), GAMRNN (CBAM and Adam), and GAMRNN (CBAM and Lion), on the CHBMIT 13-patient dataset. AUC represents the classification performance of the prediction models. As shown in the figure, the combination of the cbam module and lion optimizer has a certain effect on the classification performance of the models.

The above analysis provides a detailed examination of the individual effects of the attention module and the Lion optimizer in the proposed model. The experimental results indicate that incorporating both modules into the research on epileptic seizure prediction enhances the classification and prediction performance of the model. As shown in Figure 7, the AUC results comparison represents the model's ability to accurately classify interictal and preictal data. It can be observed that regardless of whether the Channel Attention Module (CAM) or the Spatial Attention Module (SAM) is individually integrated into the seizure prediction model or if they are combined with interchanged order, the classification performance of the model on most patients data is significantly inferior to the predictive model proposed in this study, which utilizes the Convolutional Attention Module. Figure 8 illustrates the AUC comparison of the CGRNN baseline model and the GAMRNN model, which gradually incorporates both modules, using data from 13 patients. The graph shows that the models achieve good classification performance on most patient data, which becomes more pronounced as the two modules are successively integrated. Among them, the AUC performance on the Chb01, Chb09, and Chb23 data approaches 1. However, the classification performance on the Chb02, Chb10, and Chb14 patient data is relatively lower due to the imbalance in these data categories. However, significant improvements are observed after incorporating the CBAM module and using the Lion optimizer, further demonstrating that these two modules aid in accurately recognizing and classifying imbalanced data. Therefore, the results of the above ablation experiments indicate that the proposed GAMRNN model has better EEG signal classification performance. It combines the STFT spectrogram input with channel weights, simultaneously focusing on the spatial features of the signal, and uses GRU-gated units to extract important temporal information from the features, providing specific advantages in reducing false positives and improving model accuracy.

## 5. Discussion

With the emergence of various deep learning techniques, they have gradually been applied to predict epileptic seizures. In order to compare our proposed method with other methods on the same dataset to make the comparison more convincing, we selected several studies that evaluated models using the same dataset. Table 4 shows the comparative experimental results. There is no absolute good or bad result because the models proposed by different researchers have differences, and slight changes in each step of epileptic seizure prediction may also lead to experimental differences. Our GAMRNN model is much better than the CNN model proposed by Truong et al. (2018) in all aspects. They used a three-layer convolutional model for feature extraction and achieved a prediction accuracy of 81.2% on CHB-MIT. Affes et al. (2019) proposed a CGRNN model combining three layers of convolution and two layers of gated units, achieving a classification sensitivity of 89.07%. The difference between our CGRNN model and theirs lies in the data preprocessing part, and it can be seen that the attention convolution module we introduced has a positive effect. Büyükçakır et al. (2020) utilized the Hilbert decomposition method to decompose scalp EEG data signals from 10 patients in the CHB-MIT dataset into seven components. They achieved a sensitivity of 89.8% and a false alarm rate of 0.081/h using an MLP classifier. Although our proposed method exhibits a slightly lower sensitivity, we achieved a lower false seizure prediction rate. Zhang et al. (2021) extracted the feature of multi-scale sample entropy from 23 EEG signals from the same dataset and used a bidirectional LSTM model to predict the occurrence of epileptic seizures. The prediction accuracy achieved was 80.09%, with an FPR of 0.26/h. In comparison, our model demonstrated relatively superior performance. Sun et al. (2021) also proposed a Channel Attention Dual-input Convolutional Neural Network (CADCNN) that incorporates both time-frequency spectrograms and raw EEG signals as inputs to a convolutional neural network for feature extraction and fusion. By leveraging channel attention mechanisms, their method achieved excellent results, exhibiting superior sensitivity compared to the model proposed in this study but similar AUC performance. Therefore, we hypothesize that the different forms of dual-channel input EEG signals may help improve the accuracy of feature extraction.

**Table 4:** Comparative experimental results.

**References**	**Methods**	**Accuracy**	**Sensitivity**	**FPR (/h)**	**AUC**
Truong et al. (2018)	CNN	–	81.2	0.16	–
Affes et al. (2019)	CGRNN	75.6	89.07	1.6	–
Büyükçakır et al. (2020)	MLP	–	89.8	0.081	–
Zhang et al. (2021)	Bi-LSTM	80.09	86.67	0.26	–
Sun et al. (2021)	CADCNN	–	97.1	0.029	91.7
Proposed model	GAMRNN	91.73	88.09	0.053	91.56

Our proposed study features a relatively simple overall model architecture, resulting in lower resource overhead and computational complexity. The total number of training parameters is ~880,000, including parameters from convolutional kernels, recurrent gating units, and fully connected layers. The experiments were conducted on a server equipped with an RTX 2080 Ti GPU (11 GB of VRAM), and the memory required for the dataset and model source code was ~40 GB. Training the model on the CHB-MIT dataset, which includes data from 13 patients, took ~8 h. The training time per patient varied from a few seconds to several tens of seconds per epoch, and the overall training time depended on the number of seizures and recording duration per patient. Despite its simplicity in implementation, this experiment achieved favorable performance, which highlights its relative excellence.

In the process of comparing our proposed method with others, we have reflected on potential issues that may exist. For example, the evaluation of the model on Chb02, Chb10, and Chb14 showed relatively inferior predictive performance compared to other patients. The significant inter-individual variability among patients often results in some individuals having predictable epileptic seizures while others experience unpredictable seizure occurrences. In addition to these factors, this may be closely related to the seizure condition of each patient. The Chb02 patient had only three seizures in all the records, indicating a significant imbalance in the ratio between preictal and interictal data. This imbalance adversely affected the model's ability to learn from preictal data, leading to reduced sensitivity and classification performance in identifying this data type correctly. Similarly, for the Chb10 and Chb14 patients, the relatively dense occurrence of seizure events in the recorded data files resulted in limited interictal periods available for model learning. This limitation affected the model's ability to differentiate between interictal and preictal data, leading to poorer overall classification performance. Therefore, in future research, we intend to employ data augmentation techniques to generate additional EEG data, addressing the issue of data imbalance in epileptic seizure occurrences. This endeavor aims to facilitate the epileptic seizure prediction model in achieving enhanced performance and superior outcomes.

This paper proposes a seizure prediction method based on a recurrent neural network with convolutional attention modules. Firstly, we use multiple layers of convolution to extract spatial information from multi-channel EEG recordings and apply attention mechanisms to focus on specific channels and spatial locations, mimicking the visual perception process of humans. Our model combines two channel attention modules and a spatial attention module to reassign weights to each feature channel and point in the convolution process. Two gated recurrent units are added after the attention modules to perform deep feature extraction on the temporal sequence. Experimental results show that our proposed method achieves high accuracy, sensitivity, and low false positive rate in cross-validation evaluation on the dataset, which further proves the potential of attention mechanism modules and the Lion optimization algorithm in seizure EEG prediction research, providing ideas and insights for future research in this field. In addition, we plan to explore methods for addressing imbalanced data issues and evaluate the proposed model's performance on more scalp EEG and intracranial EEG datasets to improve its generalization capability.

## Data availability statement

The datasets analyzed for this study can be found in the (CHB-MIT) (http://www.physionet.org).

## Ethics statement

The studies involving humans were approved by Clinical Investigations at the Beth Israel Deaconess Medical Center (BIDMC), Boston, Massachusetts, USA, and the Massachusetts Institute of Technology (MIT), Cambridge, Massachusetts, USA. The studies were conducted in accordance with the local legislation and institutional requirements. Written informed consent for participation was not required from the participants or the participants' legal guardians/next of kin in accordance with the national legislation and institutional requirements.

## Author contributions

WJ and HJ: conceptualization. HJ and TiX: methodology. HJ and ZY: investigation. YL: formal analysis. HJ, TiX, and TXu: writing. TXue, BC, and WJ: supervision. HJ: funding acquisition. All authors had full access to all the data in the study and take responsibility for the integrity of the data and the accuracy of the data analysis. All authors contributed to the article and approved the submitted version.
